# Effects of varicocele and microsurgical varicocelectomy on the metabolites in semen

**DOI:** 10.1038/s41598-022-08954-y

**Published:** 2022-03-25

**Authors:** Xinzong Zhang, Cuncan Deng, Wujiang Liu, Huang Liu, Yu Zhou, Qianyi Li, Houbin Zheng, Qiling Wang, Min Jiang, Tao Pang, Chunjie Ma, Cheng Huang, Qingguo Zhao, Yunge Tang

**Affiliations:** 1 NHC Key Laboratory of Male Reproduction and Genetics, Guangzhou, China; 2 Guangdong Provincial Reproductive Science Institute, 17 Meidong Road, Yuexiu District, Guangzhou, Guangdong China; 3 Department of Andrology Center of Guangdong Provincial Fertility Hospital, Guangzhou, China; 4 School of Economics and Management, Harbin Institute of Technology, Shenzhen, China; 5 Digestive Diseases Center, The Seventh Affiliated Hospital, Sun Yat-sen University, Shenzhen, China; 6 Zhaoqing West River Hospital, Zhaoqing, China

**Keywords:** Diseases, Medical research

## Abstract

The influence of varicocele and microsurgical varicocelectomy on semen quality remains unclear. Few studies have investigated the relationship between semen metabolism and the abnormalities in reproductive function caused by varicocele, however, there is no study on the changes of semen metabolism after microsurgical varicocelectomy. Here, we used the non-targeted and targeted metabolic analysis to investigate the different metabolites in seminal plasma within normal, varicocele, and varicocelectomy groups. We clearly showed that varicocele significantly affects semen metabolism, and microsurgical varicocelectomy can reverse this metabolic abnormality. Moreover, we characterized the landscape of three dipeptides in the seminal plasma of patients with varicocele that have not been identified previously in human tissues or biofluids. Interestingly, the levels of these three dipeptides decreased after microsurgical varicocelectomy coincident with an improvement in semen quality. Western blotting confirmed the downregulation of DPEP3 (dipeptidase 3) in the varicocele group and the upregulation of DPEP3 in the varicocelectomy group. Furthermore, we found that eight metabolites may be helpful to distinguish varicocele patients from normal subjects. Our results may be applied to earlier diagnosis or to predict the outcome of microsurgery for varicocele.

## Introduction

Varicocele (VCL) is a type of vascular disease that refers to the abnormal expansion, elongation and tortuosity of the veins in the spermatic cord, thus leading to pain, discomfort, and progressive testicular dysfunction. Furthermore, VCL is one of the most common causes of male infertility^1^. Previous research reported that the incidence of varicocele ranged from 10 to 15% in the male population, and from 19 to 41% in infertile males^2^. Varicocele accounts for 30% to 40% of cases involving primary male infertility and 69% to 81% of cases involving secondary infertility^3^. Due to the lack of conscious symptoms, patients often fail to receive timely diagnosis and treatment; in some patients, this can lead to an impairment of spermatogenesis^4^. Therefore, it is vital that we develop methods to diagnose and treat varicocele in an early manner.

Varicocele has negative effects on sperm concentration, motility, and morphology, and can also affect the integrity of sperm DNA^5^. Over recent years surgical treatment has become the predominant treatment method for varicocele, particularly microsurgical varicocelectomy. Microsurgical varicocelectomy can improve various aspects of sperm quality, including sperm concentration, motility and morphology^6,7^. Microsurgical varicocelectomy can also improve sperm retrieval rates in azoospermic patients suffering from varicocele and promote pregnancy and live birth rates in oligospermic patients with varicocele^8^. However, although varicocelectomy can improve sperm quality and clinical outcomes, the precise mechanisms that underlie these effects have yet to be elucidated^9^. Furthermore, the outcome of microsurgical treatment cannot be predicted effectively and despite the application of microsurgery, approximately 30% to 40% of patients show no improvements in their sperm parameters^10^.

To the best of our knowledge, few studies have investigated the relationship between sperm metabolism and the abnormalities in reproductive function caused by varicocele. Metabolomics, an important research tool for the life sciences is widely used to investigate the clinical diagnosis and pathology of disease^11^. Metabolomics analyses of plasma and blood have also been used to evaluate male infertility^12,13^. Although metabolomics has been used to evaluate male infertility and varicocele, no previous study has attempted to use metabolomics to investigate the improvement of semen parameters after microsurgical varicocelectomy^14,15^. Consequently, we know very little about the metabolic mechanisms responsible for the effects of varicocele on sperm quality or and how varicocelectomy can improve semen parameters. Furthermore, we do not yet have effective biomarkers for the prediction of such improvements.

In this study, we used ultra-performance liquid chromatography-Q Exactive Orbitrap-Mass spectrometry (UPLC-QE-MS) to carry out untargeted metabolic profiling on seminal plasma. We then analyzed and compared differences in seminal plasma between varicocele patients, the same patients after varicocelectomy, and healthy fertility controls. We compared metabolites across the three groups by correlation analysis, hierarchical clustering analysis, pathways analysis, and network analysis. Potential biomarkers were identified by receiver operating characteristic curve analysis (ROC) and multivariate linear regression. Our aim was to investigate how varicocele affects sperm metabolism and how microsurgery can improve semen parameters by influencing metabolic events.

## Patients and methods

### Patient recruitment

This study is fully in line with government policies and the Declaration of Helsinki. All experiments were approved by the Medical Ethics Committee of the Family Planning Research Institute of Guangdong Province (Reference: GDJS20200819). The study was registered on the international clinical trial registration website (https://www.researchregistry.com/browse-the-registry#home/registrationdetails/5ee9edf189de9d0015d6b185/, registration number: researchregistry5714). All patients with varicocele were willing to accept the standard microsurgical varicocelectomy through the inguinal outer ring incision, and signed the informed consent. All volunteers and varicocele patients were willing to donate their semen for the study of varicocele metabonomics. Varicocele patients or paternity-proven controls were screened for eligibility based upon inclusion and exclusion criteria listed in Supplementary Table 1. Brifly, The varicocelectomy group featured the same 30 patients as the varicocele group who accepted microsurgical varicocelectomy. Semen samples were collected 1 month before and 6 months after microsurgical varicocelectomy. The inclusion criteria were: age 18–45(years old), BMI 18.5–23.9, only left III degree varicocele, infertility for more than 1 year and no history of other congenital or acquired urogenital diseases. The exclusion criteria were: age and BMI were outside the inclusion criteria, grade I or II varicocele confirmed by B-ultrasound combined with right varicocele, obvious occupational or environmental exposure factors, history of mental diseases or unwilling to surgical treatment, combined with other congenital or acquired urogenital diseases. The normal group featured 30 age-matched normal fertile males with a history of successful fatherhood. The inclusion criteria for our healthy controls were as follows: (1) able to achieve spontaneous pregnancy and obtain live offspring; (2) sperm concentration ≥ 20 × 10^6^ spermatozoa/ml; (3) sperm progressive motility ≥ 32%; (4) normal morphology ≥ 4% and (5) no varicocele confirmed by B untrasond. All participants were fully informed and provided signed consent. In order to make the phenotypic difference more obvious, we only enrolled patients with grade III varicocele confirmed by B-ultrasound and normal controls with > 20 × 10^6^ sperm count and no varicocele confirmed by B-ultrasound. Also, the "normal" reference value of WHO version 5 has been replaced by the "critical value" of WHO version 6. Sperm concentrations were classified as "normal", "critical" and "pathological". That is, the "normal" concentration is ≥ 20 × 10^6^/ml.

### Semen specimen collection and analysis

Semen samples were obtained by masturbation (after at least 3 days of abstinence) into a sterile wide-mouth and metal-free glass container. Samples from the normal group, varicocele group, and varicocelectomy group, were stored at − 80 °C prior to use. Routine semen analysis was then carried out on a computer-aided semen analyzer (CASA) (WLJY9000, Weili New Century Technology Development Corporation, China), in accordance with guidelines described in the Fifth Edition of the World Health Organization's (WHO) Human Semen Examination Laboratory Manual. Sperm DNA integrity (DFI) was stained by acridine orange (AO) staining kit (Xindi Biocompany, Nanjing, China) and detected by BD Accuri C6 Flow Cytometry ( BD bioscience, San Jose, CA, USA). After mixing a certain amount of semen sample to be tested with sample buffer, denature it with acid solution for 30 s, then quickly add acridine orange (AO) dye for staining for 5 min, and then detect the sample by flow cytometry. Serum total testosterone was determined by human testosterone (T) ELISA kit (Mlbio, Shanghai, China), strictly followed the operation manual provided by the supplier.

### Metabolite extraction

A 100 μL aliquot of each sample was transferred to a microcentrifuge tube. Then, 400 μL of extraction solution (methanol: acetonitrile = 1:1 (v/v), containing isotopic labelled internal standard mixture) was added into each tube, and the sample was vortexed for 30 s. Then, the mixture was sonicated for 10 min in an ice-water bath and then incubated for 1 h at − 40 °Cto precipitate proteins. Next, the sample was centrifuged at 12,000 rpm for 15 min at 4°Cand the supernatant was transferred to a fresh glass vial for analysis. Quality control (QC) samples were also prepared by mixing equal amounts of supernatant from all samples.

### LC–MS/MS analysis

LC–MS/MS analyses were performed using a UHPLC system (Vanquish, Thermo Fisher Scientific, USA) and a UPLC BEH Amide column (2.1 mm × 100 mm, 1.7 μm) combined with a Q Exactive HFX mass spectrometer (Orbitrap MS, Thermo). A Thermo Q Exactive HFX mass spectrometer was used to acquire MS/MS spectra in information-dependent acquisition (IDA) mode and was controlled by acquisition software (Xcalibur, Thermo).

### Data preprocessing and annotation

First, the original data was converted into mzXML by ProteoWizard software. Then, we used R program package (kernel was XCMS) for peak recognition, extraction, alignment, and integration. Next, metabolite annotation was carried out by matching with an in-house MS database (BiotreeDB V2.1); the cutoff value for annotation was set to 0.3. We used the TIC, Mass to charge ratio(m/z) and retention time to build the model and identify the metabolites. The final dataset containing the information of peak number, sample name and normalized peak area was imported to SIMCA15.0.2 software package (Sartorius Stedim Data Analytics AB, Umea, Sweden) for multivariate analysis. Data was scaled and logarithmic transformed to minimize the impact of both noise and high variance of the variables. After these transformations, PCA (principle component analysis, PCA), an unsupervised analysis that reduces the dimension of the data, was carried out to visualize the distribution and the grouping of the samples. 95% confidence interval in the PCA score plot was used as the threshold to identify potential outliers in the dataset. In order to visualize group separation and find significantly changed metabolites, supervised orthogonal projections to latent structures-discriminate analysis (OPLS-DA) was applied. Then, a sevenfold cross validation was performed to calculate the value of R2 and Q2. R2 indicates how well the variation of a variable is explained and Q2 means how well a variable could be predicted. To check the robustness and predictive ability of the OPLS-DA model, a 200 times replacements was further conducted. Afterward, the R2 and Q2 intercept values were obtained. Here, the intercept value of Q2 represents the robustness of the model, the risk of overfitting and the reliability of the model, which will be the smaller the better. Furthermore, the value of variable importance in the projection (VIP) of the first principal component in OPLS-DA analysis was obtained. It summarizes the contribution of each variable to the model. The metabolites with VIP > 1 and *p* < 0.05 (student t test) were considered as significantly changed metabolites. In addition, commercial databases including KEGG (http://www.genome.jp/kegg/) and MetaboAnalyst (http://www.metaboanalyst.ca/) were used for pathway enrichment analysis.

### Targeted metabolic analysis and western blotting

Standard tyrosyl-phenylalanine was purchased from Sigma-Aldrich (St. Louis, MO, USA). Standards for tyrosyl-Isoleucine and leucyl-gamma-glutamate were synthesized by GenScript (Nanjing) Co., Ltd. (Nanjing, China). Semen samples from 30 controls with confirmed fertility and 30 varicocele patients including preoperative and postoperative semen were used for DHPLC mass spectrum analysis. The same samples were also used for western blotting to detect DPEP3. RIPA buffer was used to lyse the cells and total protein was extracted. The protein concentration was determined by a BCA Protein Assay Kit ( Thermo Fisher Scientific, Shanghai, China). An appropriate amount of each protein sample was denatured and separated by 10% SDS-PAGE (polyacrylamide gel electrophoresis). Separated proteins were then transferred to a Polyvinylidene fluoride (PVDF) membrane, blocked for 1 h, washed with TBST buffer, and then incubated at 4 °C overnight with a primary antibody against DPEP3 or GAPDH antibody (1:1,000; Abcam, Cambridge, MA, USA). The following morning, membranes were washed with TBST ( Tris Buffered Saline with Tween® 20) buffer, and incubated with a secondary antibody (1:2,000) at room temperature for 1 h. The expression levels of DPEP3 were then calculated with GAPDH as internal reference. The experiment was repeated three times independently. All the samples were assessed separately.

### Statistical analysis

The clinical characteristics of varicocele group and normal group were statistically analyzed by the Student's t-test.and Mann–Whitney’s U test. Multidimensional statistical analyses of metabolites were performed using MetaboAnalyst version 4.0 (http://www.metaboanalyst.ca), and included unsupervised principal component analysis (PCA) and supervised partial least squares discriminant analysis (PLS-DA). Differential metabolites were determined by creating volcano plots with a variable importance (VIP) threshold > 1 and a *t-*test threshold < 0.05. Then, we used receiver operating characteristic (ROC) curve analysis to identify differential metabolites and candidate biomarkers. Multiple linear regression analysis was also performed to discover the correlation between different semen parameters (including concentration and motility) and potential biomarkers. SPSS (version 20.0; SPSS IBM Corp., Armonk, NY) was used for all statistical analyses. P values less than 0.05 were regarded as statistically significant.

## Results

### Clinical characteristics of the participants and outcomes

The ages and BMI (Body Mass Index) of patients in the fertile control group were matched with those of the varicocele group and the varicocelectomy group (*P* > 0.05). The sperm concentration in the control group (58.30 ± 22.33 × 10^6^/mL) was significantly higher than that in the varicocele group (25.00 ± 29.81 × 10^6^/mL). The progressive motility of sperm in patients from the varicocele group (29.09% ± 12.89) was significantly lower than that in the control group (57.97% ± 11.45). Both sperm concentration and motility were significantly improved in patients from the varicocelectomy group than the varicocele group (*P* < 0.05). All clinical data are shown in Table 1.

**Table 1:** Clinical characteristic of the participants.

	Normal group	Varicocele group	Varicocelectomy group
Number	30	30	30
Age	32.07 ± 3.93	32.10 ± 4.15	32.73 ± 4.14
BMI	22.05 ± 1.91	21.79 ± 1.88	21.79 ± 1.88
Smoking (yes/all)	0/30	0/30	0/30
Alcohol (yes/all)	0/30	0/30	0/30
Varicocele degree	no	III (only left)	III (only left)
Left testicular volume(cm^3^)	19.79 ± 3.26	14.18 ± 1.69	14.18 ± 1.69
Right testicular volume(cm^3^)	20.81 ± 3.58	15.43 ± 3.45	15.43 ± 3.45
Sperm concentration	58.30 ± 22.33	25.00 ± 29.81^a^	40.95 ± 54.04^b^
Progressive motility (PR, %)	57.97 ± 11.45	29.09 ± 12.89^a^	46.49 ± 14.96^b^
Total motility (TM, %)	75.40 ± 8.78	38.12 ± 13.73 ^a^	60.76 ± 15.91^b^
Normal morphological sperm (%)	8.41 ± 3.25	2.00 ± 1.00^a^	3.98 ± 2.38
DNA fragmentation index	12.03 ± 2.96	19.33 ± 2.75^a^	14.16 ± 2.23^b^
Serum testosterone(nmol/L)	19.12 ± 4.08	14.65 ± 3.96	16.27 ± 3.74

^a^Compared with the control group, *P* < 0.05.

^b^compared with the varicocele group, *P* < 0.05.

### Differential metabolites and volcano mapping

**Figure 1:**
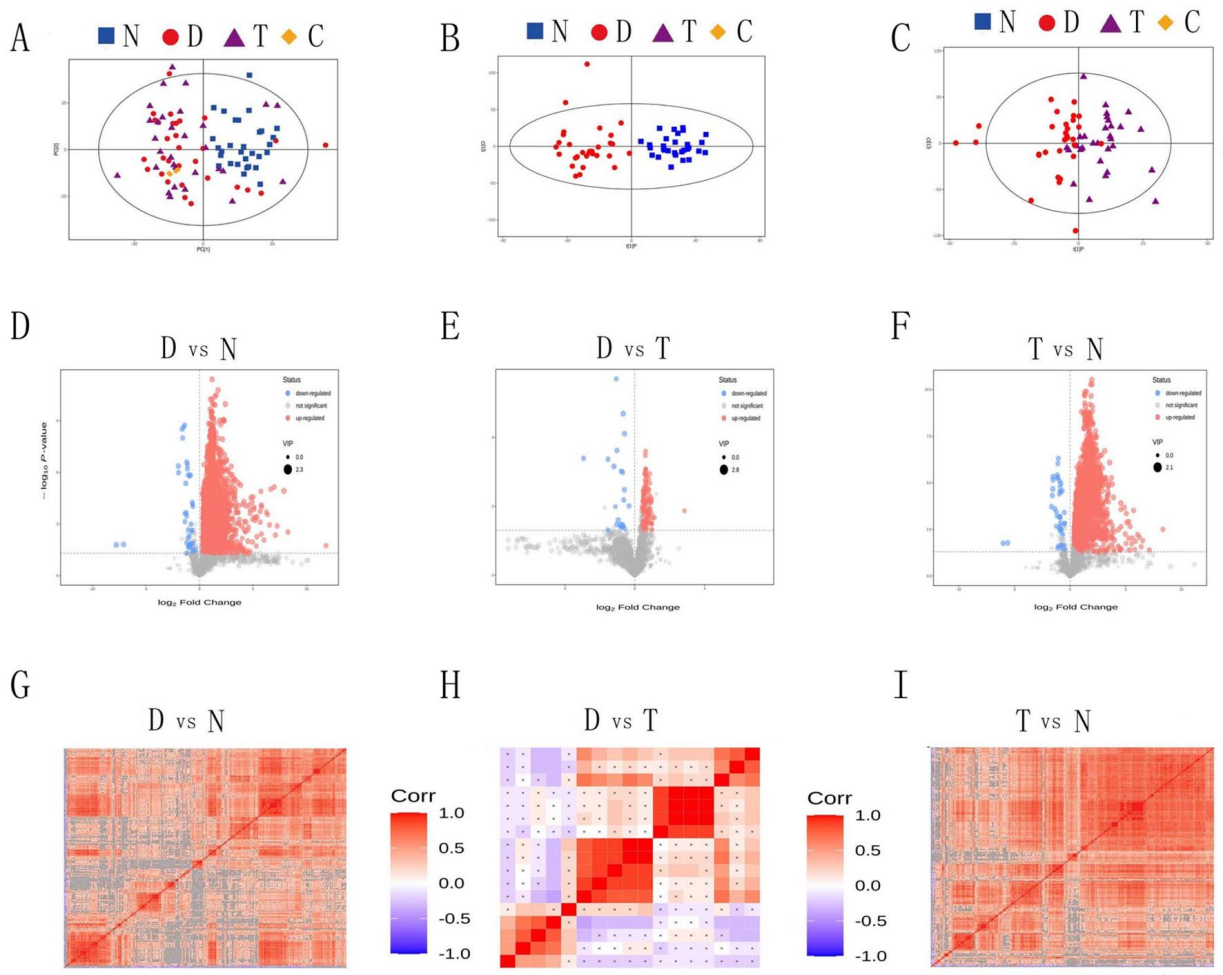
(**A**, **B**, **C**): Quality control of metabolomics analysis. Panel A shows a PCA(Principal Component Analysis)score scatter diagram for all samples (including the QC sample).The abscissa PC^1^ and ordinate PC^2^ represent the scores of the first and second principal components, respectively; the scatter color and shape represent the experimental groups of samples. Panels B and C show the OPLS-DA(Orthogonal Partial Least Squares Discrimination Analysis) scatter chart. The abscissa t^1^ P represents the predicted principal component score of the first principal component, the ordinate t^1^ O represents the orthogonal principal component score, and the scatter shape and color represent different experimental groups. N: normal control group, D: varicocele group, T: varicocelectomy group, C: quality control group.PCA and OPLS-DA were generated by SIMCA software and the version is 16.0.2. (**D**, **E**, **F**): Differentially expressed metabolites represented in the form of volcano plots. Each point represents a metabolite. The abscissa represents multiple changes of the group compared with each substance (the logarithm based on 2), the ordinate represents the p value from the Student's *t-*test (the negative logarithm was based on 10), and the scatter size represents the VIP value of the OPLS-DA mode. The larger the scatter, the greater the VIP value. The scatter color represents the final screening result. The significantly up-regulated metabolites are shown in red, the significantly down-regulated metabolites are shown in blue, and the metabolites without a significant difference are shown in gray. N: normal control group, D: varicocele group, T: varicocelectomy group. The Volcano Plot is generated by R(ggplot2) software and the version is 3.3.5. (**G**, **H**, **I**): Correlation analysis of differentially expressed metabolites. The abscissa and ordinate represent a comparison of the differentially expressed metabolites between groups. The color blocks in different positions represent the correlation coefficient between the metabolites at corresponding positions: red indicates positive correlation, and blue indicates negative correlation. Insignificant correlation is shown as a cross. N: normal control group, D: varicocele group, T: varicocelectomy group. The Correlation Heatmap is generated by R(corrplot) and the version is 0.89.

The differential metabolites that were identified by this analysis are shown in Fig. 1D–F in the form of a volcano plot and shows that there were significant differences in the levels of semen metabolites when compared between the controls, varicocele, and varicocelectomy groups. Compared with the control group, 275 differential metabolites were identified between the varicocele group and the control group. There was also a difference in the levels of semen metabolites after surgery compared to levels before the surgery, but this difference was significantly smaller than when compared to the fertile controls. This suggested that the levels of metabolites may not be fully restored to normal levels following surgery.

### Hierarchical cluster analysis of differential metabolites

**Figure 2:**
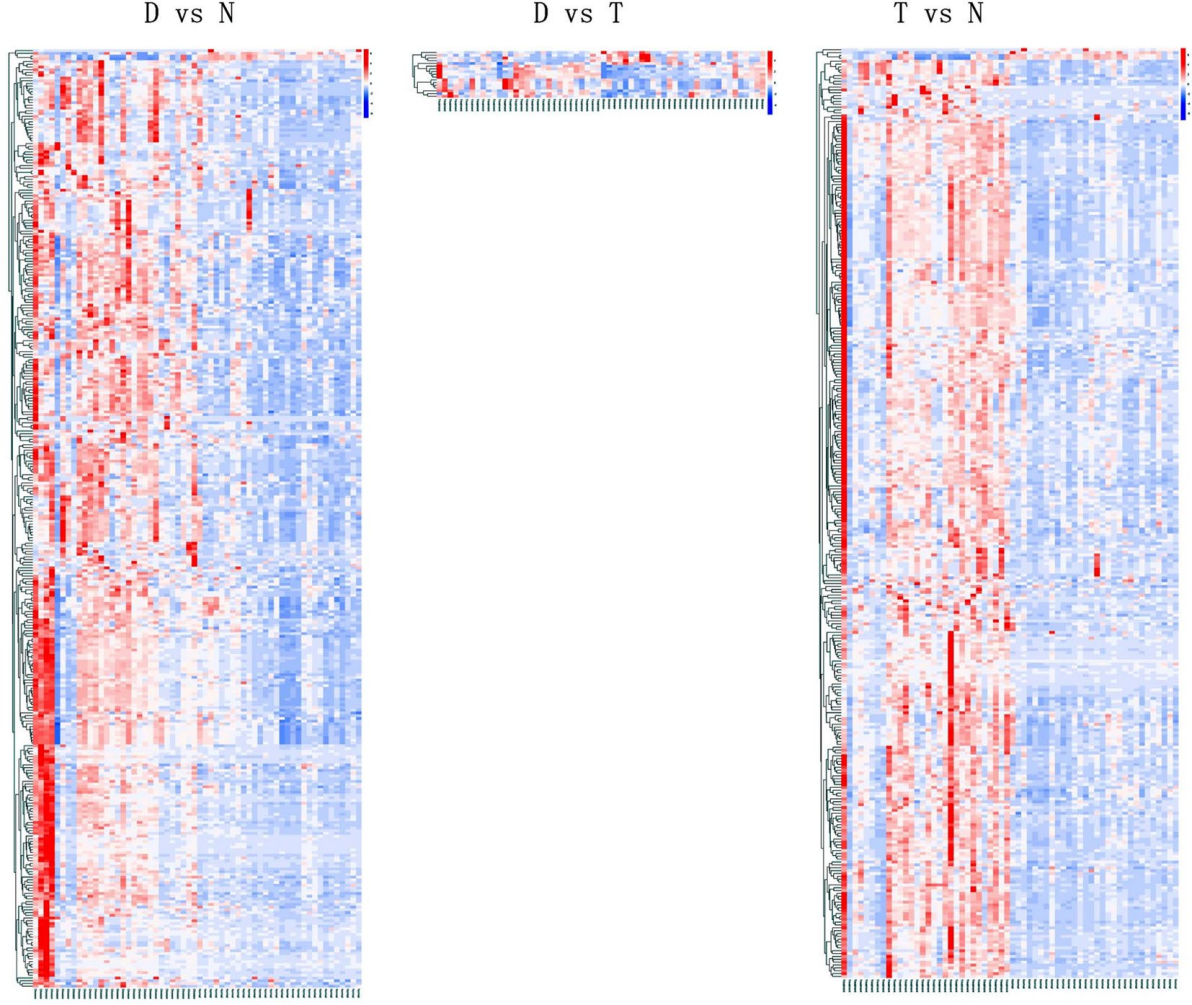
Heat map of hierarchical clustering analysis. The abscissa represents different experimental groups, the ordinate represents the differentially expressed metabolites by group, and the color blocks at different positions represent the relative expression of the corresponding metabolites. N: normal control group, D: varicocele group, T: varicocelectomy group. The Hierarchical clustering heatmap is generated by R(pheatmap) and the version is 1.0.12.

Figure 2 shows the hierarchical clustering of the top 50 significant different metabolites among the control, varicocele, and varicocelectomy groups. These results showed that there was a certain trend for clustering for several differential metabolites when considered across the three groups. There was also a clustering trend for certain differential metabolites between the varicocelectomy group and varicocele groups; however, this difference was significantly smaller than between the varicocele group and the control group.

### Pathway analysis of different metabolites

**Figure 3:**
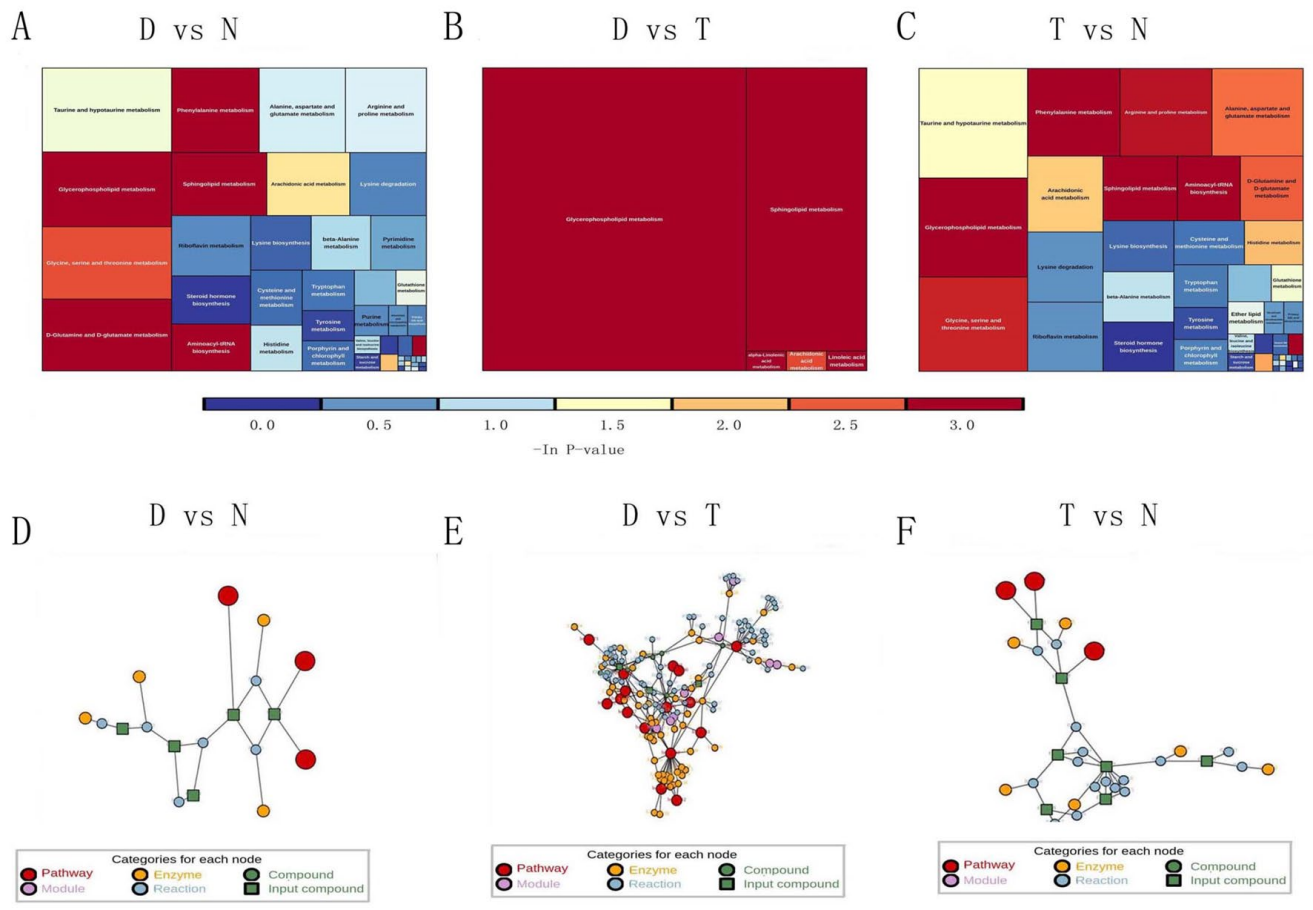
(**A**, **B**, **C)**: Pathway analysis of different metabolites. The results of metabolic pathway analysis are shown as a rectangular tree. Each block in the rectangular tree represents a metabolic pathway, and the block size represents the influential factors of the pathway in the topological analysis. The larger the block size, the larger the influential factor; the block color represents the p value of enrichment analysis (taking a negative natural logarithm, i.e. -ln (P)). The darker the color, the smaller the P value, and the more significant the degree of enrichment. N: normal control group, D: varicocele group, T: varicocelectomy group. (**D**, **E**, **F**): Regulatory network analysis of differentially expressed metabolites. The red dot represents a metabolic pathway, the yellow dot represents a substance related to a regulatory enzyme, the green dot represents the background material of a metabolic pathway, the purple dot represents the molecular module (class), the blue dot represents a chemical interaction reaction, and the green dot represents the differentially expressed substances. N: normal control group, D: varicocele group, T: varicocelectomy group. The treemap plot is generated by R(KEGGgrph,treemap) software and the version is 1.46.0, 2.4–2. The network plot is generated by R(network,igraph) software and the version is 1.16.1,1.2.6.

The pathways associated with 275 differentially expressed metabolites between the varicocele group and the control group are shown in Fig. 3A–C. These 275 differential metabolites were associated with 39 pathways in patients with varicocele. Pathway analysis further indicated that varicocele influences semen quality via taurine and hypotaurine metabolism, amino acid metabolism, glycerol phospholipid metabolism, sphingolipid metabolism, et al. Interestingly, only 17 metabolites were involved in metabolic events involving sphingolipid and glycerophospholipid metabolism, when compared between the varicocelectomy and varicocele groups. This suggested that the improvement of semen quality after microsurgery may be related to the metabolism of glycerophosphate and sphingolipid (Fig. 3A–C).

### Regulatory network analysis for differential metabolites

After acquiring matching information related to different metabolites, we searched the KEGG database for *Homo sapiens* (human) and analyzed the network of regulatory interactions^16–18^. Data arising from this regulatory analysis are shown in a network plot (Fig. 3D–F). Although the differences of metabolites between the varicocelectomy and varicocele group were relatively small, the differential metabolites were associated with more complex regulatory networks (Fig. 3), thus implying the importance of these differential metabolites.

### Potential biomarkers and their correlations with semen parameters

**Figure 4:**
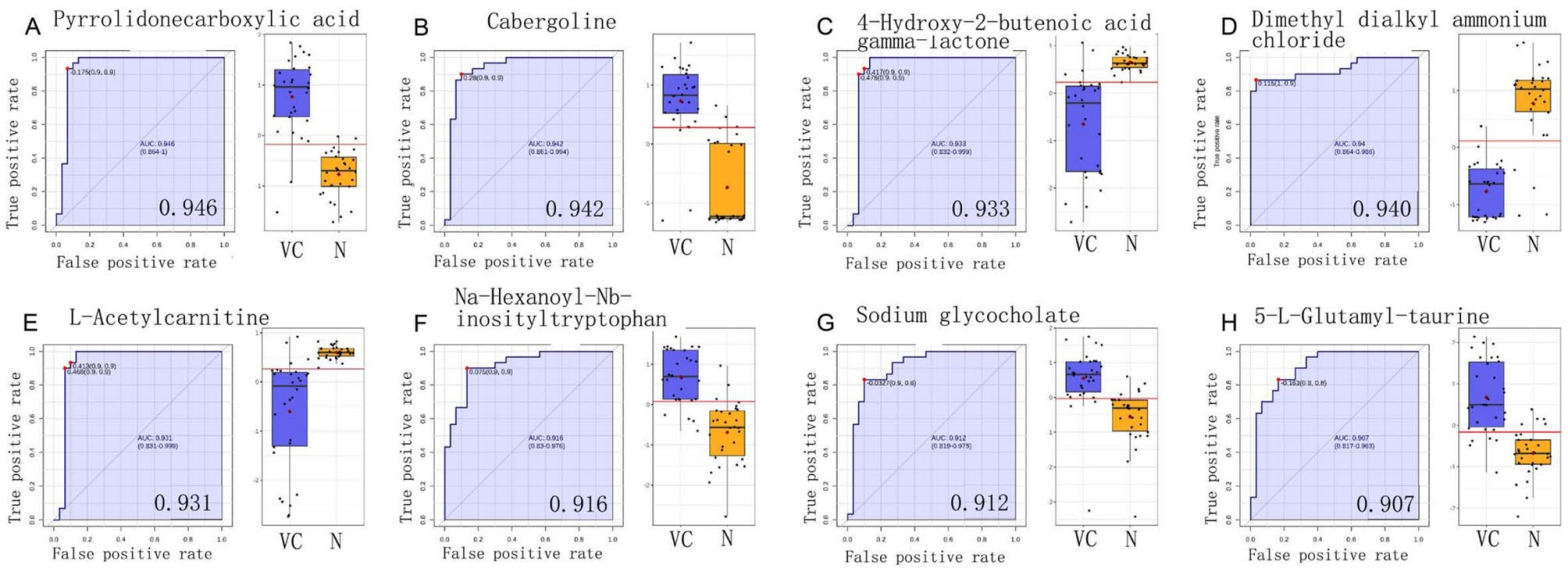
Potential biomarker analysis. The horizontal axis shows the false positive rate (= 1-specificity). The vertical axis shows true positive rate (= sensitivity).AUC = area under curve; N: normal control group, VCL: varicocele group.

**Figure 5:**
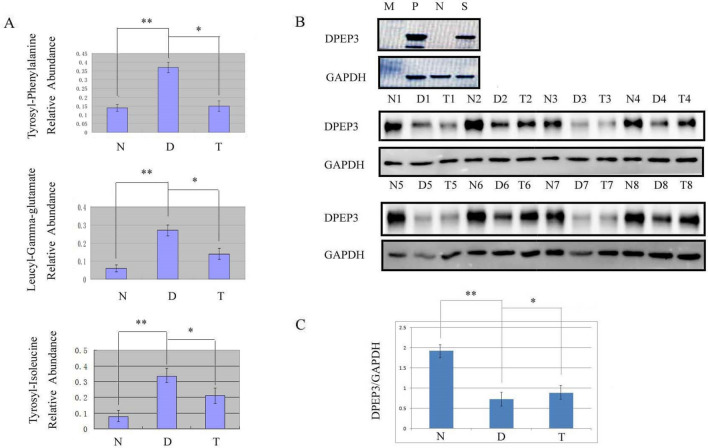
Dipeptide metabolism of sperm and seminal plasma. Panel A shows the relative abundance of the three dipeptides confirmed by targeted metabonomics. Panel B shows representative western blots for DPEP3 expression among the normal control group, varicocele group and varicocelectomy group. Blots were distributed on three different gels. Electrophoresis, hybridization and exposure were performed under the same conditions. The intensity of the bands was analyzed by Image J software, and t-test was used for statistical analysis. Panel C shows statistical results for each group. P: positive control, N: negative control, S: sample, N: normal control group, D: varicocele group, T: varicocelectomy group.^**^P < 0.01; ^*^P < 0.05.

Next, we performed receiver operating characteristic curve (ROC) analysis for all metabolites. With an area under the curve (AUC) threshold > 0.9, we identified eight that may represent potential biomarkers, including pyrrolidonecarboxylic acid, cabergoline, 4-hydroxy-2-butenoic acid-gamma-lactone, dimethyl dialkyl ammonium chloride, L-acetylcarnitine, Na-Hexanoyl-Nb-inosityltryptophan, Sodium glycocholate and 5-L-Glutamyl-taurine (Fig. 4 and Supplementary Table 2). Using a threshold of *P* < 0.05, no metabolites that were selected by the ROC curves were found to be correlated with sperm concentration. The 5-L-Glutamyl-taurine was significantly positively correlated with motility (*P* < 0.05) while other biomarkers were not correlated with motility. Interestingly, three dipeptides, including leucine-dental-glutamate, tyrosyl-phenylalanine, and tyrosyl-isoleucine, were identified for the first time in human seminal plasma. These were significantly increased in the seminal plasma of patients with varicocele (*P* < 0.01). The quality of semen recovered, and the level of dipeptides, decreased after surgery. These dipeptides represent potential biomarkers for varicocele and useful biomarkers for predicting surgical outcome. The relative abundance of three dipeptides were further confirmed by targeted metabolomics (Fig. 5A).

### DPEP3 was downregulated in the varicocele group but upregulated in the varicocelectomy group.

Next, we used western blotting to compare the levels of DPEP3, a key enzyme involved in testicular dipeptide metabolism, in the normal group, varicocele group, and varicocelectomy group. The relative abundance of DPEP3 was significantly lower in the varicocele group than that in the normal group (*P* < 0.01) and the varicocelectomy group (*P* < 0.05) (Fig. 5C).

## Discussion

In this study, we performed UHPLC-QE-MS untargeted metabolomics to detect metabolites in the seminal plasma from controls, varicocele and varicocelectomy groups. Our results clearly show that varicocele significantly affects sperm metabolism, and microsurgical varicocelectomy can reverse this metabolic abnormality. We then identified potential biomarkers to discriminate varicocele from normal controls and to predict the microsurgical outcomes, thus providing tools that can be used to diagnose varicocele. We also identified that certain dipeptides existed in the metabolites of seminal plasma in patient with varicocele; a reduction in the levels of these dipeptides may contribute to the improvement of semen quality after microsurgery.

The PCA scroe chart showed that the sample quality was good, the experimental methodology was excellent, and the system stability control was outstanding. All the sample is basically within the 95% confidence interval (Hotelling's t-squared ellipse) (Fig. 1A). It can be seen from the results of OPLS-DA score chart that the difference between the normal and the varicocele groups of samples is very significant (Fig. 1B), and the difference between the varicocele and the varicocelectomy groups of samples is also very significant(Fig. 1C). We also used OPLS-DA replacement test to avoid over fitting of the test model and evaluate the statistical significance of the model. The Q2 value of the random model of replacement test is less than that of the original model; The intercept between the regression line of Q2 and the longitudinal axis is less than zero; At the same time, with the gradual decrease of replacement retention, the proportion of Y variables of replacement increases, and the Q2 of random model decreases gradually. It shows that the original model has good robustness and there is no over fitting phenomenon.

Although metabonomics has been applied to the study of male infertility^14–23^, so far, it has not been applied to the study of varicocelectomy, especially the change of semen quality after microsurgery. As shown in Fig. 1D–F and Supplementary Table 2, there were marked differences in terms of metabolites in the seminal plasma when compared between the controls, varicocele, and varicocelectomy groups. The reason for this great difference may be that the blood flow is affected by varicocele, resulting in local ischemia and anoxia of testis, and the metabolism of sperm is decreased. Whatever, these results hint that the determination of sperm metabolic changes may be used as a tool for the diagnosis and treatment of varicocele^24^. Our results showed that differences in the levels of metabolites before and after microsurgery were significantly reduced compared with those in patients with varicocele and controls, although there was still strong correlation and clustering properties (Fig. 1G–I). The main reason for these findings may be related to individual genetic background. We compared the same patients before and after surgery; in contrast, patients with varicocele and controls were entirely different individuals. Neto FTL, et al . studied the changes of semen metabolism in varicocele by using nuclear magnetic resonance technology^14^. Similar to our conclusion, varicocele can indeed cause significant differences in semen metabolites, but the molecular marker they found is different from what we found. Except for the differences in individual samples, it may be related to the experimental methods used and the high variability of metabolic small molecules.

The differential metabolites obtained by analysis often have biological results and functional similarity / complementarity, or are positively / negatively regulated by the same metabolic pathway, showing similar or opposite expression characteristics among different experimental groups. Hierarchical cluster analysis of these characteristics helps us to classify metabolites with the same characteristics into one category and identify the change characteristics of metabolites between experimental groups. For each group of comparison, we use the quantitative value of differential metabolites to calculate the Euclidean distance matrix, cluster the differential metabolites by complete linkage method, and display them by thermodynamic diagram. It can be seen from the figure that the normal group, varicocele group and varicocelectomy group have significantly different clustering characteristics (Fig. 2). It is particularly noteworthy that the clustering between the operation group and the varicose vein group, although there are few different metabolites, still shows significantly different clustering characteristics, and the clustering characteristics of the varicocelectomy group are close to those of the normal group. This shows that the operation does have a positive impact on sperm metabolism.

Our results also showed that the metabolism of glycerophospholipid and sphingolipid were the main changes in sperm metabolism following surgery, and that the main metabolic molecules in these two pathways were significantly up-regulated after microsurgery (Fig. 3A–C). Diphosphatidylglycerol, one of the main phospholipids synthesized by the mitochondrial inner membrane, is related to the assembly and activity of many protein complexes in the mitochondria^25^. These results suggested that the increased mitochondrial activity of sperm may be related to an increase of sperm energy supply and motility. Sphingolipid and its metabolites are not only important structural molecules for the cell membrane, they are also involved in many important signal transduction processes, including cell growth, differentiation, senescence, and programmed cell death^26^. This may explain the improvement of sperm morphology (membrane structure), the reduction in sperm apoptosis, and the increase in sperm concentration, following surgery. Although the differences in metabolic molecules before and after microsurgery was relatively small, these differentially expressed molecules were associated with complex molecular network regulation, thus indicating the importance and influence of these differentially expressed molecules (Fig. 3D–F).

8 metabolites and 3 dipeptides were identified to be potential biomarkers for varicocele (Fig. 4 and 5). Although it is easy to diagnose varicocele by touch and B-ultrasound, the early diagnosis of varicocele and whether it should be operated are still difficult. Using ROC analysis, we found that 8 metabolites had a good differentiation between varicocele group and normal group, and AUC was more than 90% (Fig. 4). However, due to the instability and variability of metabolic small molecules, it is still difficult to use a single small molecule for VC diagnosis. In the future, we plan to explore the possibility of combined application of these 8 metabolites as VC diagnosis. Interesting, 3 dipeptides have not been identified in any other biological fluid before. We found that these dipeptides existed in serum of varicocele. In patients with varicocele, the concentration of these dipeptides decreased after operation, but still existed. However, the dipeptides were not detectable in the normal group of males. It was also difficult to detect these dipeptides in the semen of vasectomized patients, indicating that these dipeptides are not derived from the prostate, seminal vesicles, or other accessory gonads (data not shown). Further, through the method of targeted metabolomics, we confirmed that there was a very significant difference between 30 normal semen samples and 30 semen samples from patients with varicocele (Fig. 5A). Therefore, we believe that the dipeptide in the semen of patients with varicocele may be a biomarker of varicocele, However, whether it can be applied to early diagnosis or prediction of surgical effect is still under investigation.

We also found that DPEP3 was expressed in sperm and that levels of this dipeptidase were reduced in varicocele patients. Intersting, after surgery, this dipeptidase inceased with decrased dipeptide [Fig. 5B, C]. DPEP3 gene encodes a membrane-bound glycoprotein from the family of dipeptidases involved in hydrolytic metabolism of various dipeptides. Although DPEP3 mRNA was detected in testis (× 39.8 times) and whole blood (× 7.4 times), its protein was only detected in ovary (×54.4 times) and testis (×10.4 times). Therefore, we assume that dpep3 plays a very important role in degrading dipeptides in semen of patients with varicocele. So far, we still know little about the amino acid utilization and amino acid metabolism in sperm. However, clinically, there are infertile patients who have improved semen quality by taking compound amino acid capsules. Also, a reduction in the levels of leucine will result in a reduction in antioxidant and anti-inflammatory ability^27^. Furthermore, a reduction of glutamic acid will lead to a reduction in acid resistance, thus making sperm vulnerable to damage^28^. Therefore, the changes of amino acid profile in semen may affect sperm metabolism and semen quality. How DPEP3 regulates the dipeptide level and amino acid profile in semen of patients with varicocele remains to be further studied. Manesh Kumar Panner Selvam et al. studied the changes of semen protein content in varicocele with proteomics technology^15^. Similar to our conclusion, varicocele can indeed cause significant changes in semen protein content, but DPEP3 is not among the differential proteins they found, which may be related to the different sensitivity of experimental methods and protein abundance.

### Limitations

The disadvantage of this study is that it is a single center, small sample study. Only the left grade III varicose veins are selected for the study. The selected biomarkers need to be further confirmed in large samples and different stages of disease development.

## Conclusion

In this study, we first used UHLPC-QE-MS-untargeted metabolomics to detect metabolites in the seminal plasma from varicocele patients and patients receiving microsurgical varicocelectomy. The small metabolic molecules in the semen of patients with varicocele are significantly different from those of normal fertile people, and the small metabolic molecules of the same patient also change significantly before and after varicocelectomy, which proves that the varicocelectomy can improve the semen quality by changing the sperm or semen metabolism. At the same time, it was found that 8 metabolites and 3 dipeptides may be used as biomarkers of varicocele. Further study found that DPEP3, the key enzyme of dipeptides degradation, had significant changes in normal group, varicocele group and varicocelectomy group. The changes of semen metabolism before and after varicocelectomy mainly involve glycerol phospholipid metabolism, sphingolipid metabolism and amino acid metabolism. It is expected to screen the key molecules affecting semen quality from these metabolic pathways in the future. Further studies are now needed to further elucidate the mechanisms underlying varicocele and how microsurgical varicocelectomy can improve semen quality.

## Supplementary Information

Supplementary Information.
