# Supplementary material for: Effects of varicocele and microsurgical varicocelectomy on the metabolites in semen
Source: Sci Rep. 2022 Mar 25;12:5179. doi: 10.1038/s41598-022-08954-y (PMC8956711; doi:10.1038/s41598-022-08954-y)
Supplement: Supplementary file 1 — Supplementary Information. [file 41598_2022_8954_MOESM1_ESM.pdf]

Supplementary Table 1: Inclusion and exclusion criteria of the participants

Patient information	Inclusion criteria	Exclusion criteria
Age (year)	18-45	
BMI (Body Mass Index)	18.5-23.9	
Varicocele degree	III(only left)	I, II or Right
Years of sterility	⩾1	<1
Microsurgical varicocelectomy	accept	decline
Scrotal surgery	-	+
Testicular biopsy	-	+
Cryptorchidism	-	+
Testicular torsion	-	+
Occupational exposure	-	+
Environmental toxic substances	-	+
Serious physical and mental diseases	-	+
Hydrocele of the tunica vaginalis	-	+
Scrotal masses	-	+
Urinary anomalies	-	+

PS:

The varicocelectomy group featured the same 30 patients as the varicocele group who accepted microsurgical varicocelectomy. Semen samples were collected 6 months after microsurgical varicocelectomy. The normal group featured 30 age-matched normal fertile males with a history of successful fatherhood. The inclusion criteria for our healthy controls were as follows: (1) able to achieve spontaneous pregnancy and obtain live offspring; (2) sperm concentration $\geq 20 \times 10^6$ spermatozoa/ml; (3) sperm progressive motility ≥32%; and (4) normal morphology ≥4%. All participants were fully informed and provided signed consent.

Supplementary Table 2: Statistical analysis of important differentially expressed metabolites

Metabolites	Varicocele *vs* Normal		Varicocelectomy *vs* Varicocele	
	Fold-change	P-value	Fold-change	P-value
Tyrosyl-Phenylalanine	2.136	0.002	0.611	0.035
Tyrosyl-Isoleucine	4.175	2.833E-5	0.629	0.038
Leucyl-Gamma-glutamate	3.203	0.002	0.518	0.025
pyrrolidonecarboxylic acid	3.916	1.606E-9	0	0
cabergoline	28.144	3.065E-5	0	0
4-Hydroxy-2-butenoic acid gamma-lactone	0.438	2.540E-7	0	0
dimethyl dialkyl ammonium chloride	0.007	0.015	0	0
L-acetylcarnitine	0.483	1.399E-6	0	0
Na-Hexanoyl-Nb-inosityltryptophan	4.606	3.211E-7	0	0
Sodium glycocholate	8.307	4.157E-6	0	0
5-L-Glutamyl-taurine	7.290	1.438E-6	0	0
N-[(4E,8Z)-1,3-dihydroxyoctadeca-4,8-dien-2-yl]hexadecanamide 1-glucoside	1.994	3.241E-5	1.467	0.007
PC(P-16:0/18:4(6Z,9Z,12Z,15Z))	0	0	1.813	0.008
N-Lignoceroylsphingosine	0	0	0.798	0.010
N-Palmitoylsphingosine	2.330	2.914E-8	1.433	0.004
PC(15:0/24:0)	2.094	6.640E-6	1.552	0.002
Fluticasone propionate	3.581	0.002	0.492	0.034
SM(d18:0/18:1(9Z))	2.382	2.325E-9	1.433	0.008
SM(d17:1/24:1(15Z))	2.495	1.169E-7	1.423	0.013
SM(d18:1/20:0)	2.371	2.228E-8	1.419	0.006
Cer(d18:0/18:1(11Z))	3.065	5.054E-5	1.423	0.045
SM(d18:1/24:1(15Z))	2.537	1.101E-8	1.405	0.016
Glucosylceramide (d18:1/18:0)	1.999	2.575E-6	1.387	0.007
N-[(4E,8E)-1,3-dihydroxyoctadeca-4,8-dien-2-yl]hexadecanamide	2.182	3.898E-4	1.590	0.007
PC(P-18:1(9Z)/14:0)	1.997	1.888E-5	1.478	0.004

Supplementary Table 3: Software and Version used in the muanuscript

Analysis	Software	Version
PCA	SIMCA	(16.0.2)
OPLS-DA	SIMCA	(16.0.2)
Permutation plot test of OPLS-DA	R(ggplot2)	(3.3.5)
Volcano plot	R(ggplot2)	(3.3.5)
Hierarchical clustering heatmap	R(pheatmap)	(1.0.12)

Correlation Heatmap	R(corrplot)	(0.89)
treemap plot	R(KEGGgrph,treemap)	(1.46.0, 2.4-2)
network plot	R(network,igraph)	(1.16.1,1.2.6)

Ps: The original code used by pheatmap comes from
https://github.com/raivokolde/pheatmap.

Supplementary file 4: informed consent

All patients with varicocele were willing to accept the standard microsurgical varicocele ligation through the inguinal outer ring incision, and signed the informed consent. All volunteers and varicocele patients were willing to donate their semen for the study of varicocele metabonomics.

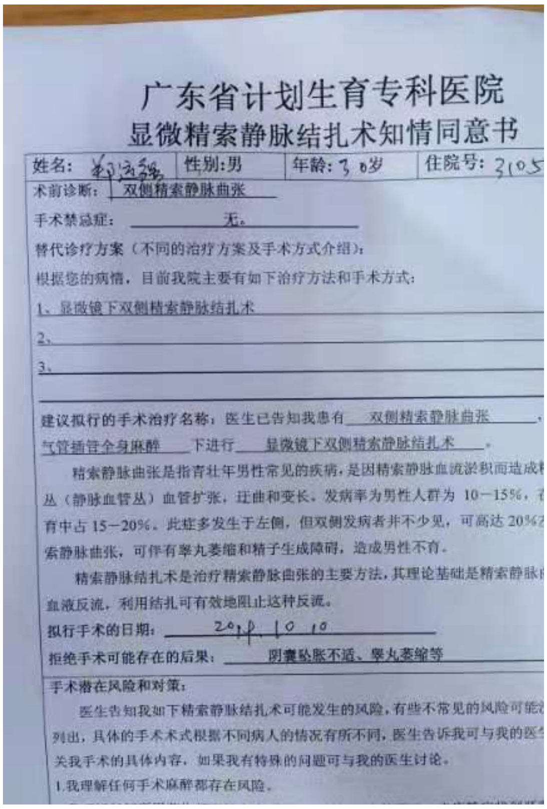

广东省计划生育专科医院
显微精索静脉结扎术知情同意书

姓名：[illegible]	性别:男	年龄：30岁	住院号：3105

术前诊断：双侧精索静脉曲张

手术禁忌症：无。

替代诊疗方案（不同的治疗方案及手术方式介绍）：

根据您的病情，目前我院主要有如下治疗方法和手术方式：

1、显微镜下双侧精索静脉结扎术

2、

3、

建议拟行的手术治疗名称：医生已告知我患有 双侧精索静脉曲张，
气管插管全身麻醉 下进行 显微镜下双侧精索静脉结扎术。

精索静脉曲张是指青壮年男性常见的疾病，是因精索静脉血流淤积而造成[illegible]
丛（静脉血管丛）血管扩张，迂曲和变长。发病率为男性人群为 10－15%，[illegible]
育中占 15－20%。此症多发生于左侧，但双侧发病者并不少见，可高达 20%[illegible]
索静脉曲张，可伴有睾丸萎缩和精子生成障碍，造成男性不育。

精索静脉结扎术是治疗精索静脉曲张的主要方法，其理论基础是精索静脉[illegible]
血液反流，利用结扎可有效地阻止这种反流。

拟行手术的日期：2018.10.10

拒绝手术可能存在的后果：阴囊坠胀不适、睾丸萎缩等

手术潜在风险和对策：

医生告知我如下精索静脉结扎术可能发生的风险，有些不常见的风险可能[illegible]
列出，具体的手术术式根据不同病人的情况有所不同，医生告诉我可与我的医[illegible]
关我手术的具体内容，如果我有特殊的问题可与我的医生讨论。

1.我理解任何手术麻醉都存在风险。

3) √损伤周围脏器(肠管，膀胱，神经等)；
4) √术中情况改变术式；
5) √术后伤口感染，伤口愈合不良，脂肪液化，伤口疤痕形成，切口[illegible]
6) √深静脉血栓形成；
7) √精液质量无明显改善，局部症状无缓解，精索静脉曲张复发，无法[illegible]
8) √睾丸鞘膜积液，阴囊水肿。

4.我理解如果我患有高血压、心脏病、糖尿病、肝肾功能不全、静脉血栓[illegible]吸烟史，以上这些风险可能会加大，或者在术中或术后出现相关的病情加[illegible]意外，甚至死亡。

5.我理解术后如果我的体位不当或不遵医嘱，可能影响手术效果。

患者存在的特殊风险或主要高危因素

我理解根据我个人的病情，我可能出现以下特殊并发症或风险：

一旦发生上述风险和意外，医生将以高度的责任心，认真执行手术操[illegible]救物品的准备及手术过程中的监测，针对可能发生的并发症做好应对措[illegible]术意外或并发症，我们将积极采取相应的抢救措施。但由于医疗技术水平[illegible]人体质的差异，意外风险不能做到绝对避免，且不能确保救治完全成功，[illegible]疾、组织器官损伤导致功能障碍，甚至死亡等严重不良后果及其他不可预[illegible]的的特殊情况，恳请理解。

患者知情选择

- 我的医生已经告知我的病情和医疗措施，需要实施的手术、特殊[illegible]疗、医疗风险、替代医疗方案等情况。
- 我已了解可能存在的其它治疗方法，选择了显微镜下精索静脉[illegible]了解手术可能存在的风险、效果及预后等情况，并知道手术是创[illegible]由于受医疗技术水平的局限、个体差异的影响，术中术后可能发[illegible]
[illegible]
